# *In vitro *interactions of *Candida parapsilosis *wild type and lipase deficient mutants with human monocyte derived dendritic cells

**DOI:** 10.1186/1471-2180-11-122

**Published:** 2011-05-29

**Authors:** István Nagy, Kata Filkor, Tibor Németh, Zsuzsanna Hamari, Csaba Vágvölgyi, Attila Gácser

**Affiliations:** 1 Institute for Plant Genomics, Human Biotechnology and Bioenergy, Bay Zoltán Foundation for Applied Research, Derkovits fasor 2., 6726 Szeged, Hungary; 2 Department of Microbiology, University of Szeged, Közép fasor 52, H-6726 Szeged, Hungary

**Keywords:** *Candida*, dendritic cell, innate immunity, secreted lipase

## Abstract

**Background:**

*Candida parapsilosis *typically is a commensal of human skin. However, when host immune defense is compromised or the normal microflora balance is disrupted, *C. parapsilosis *transforms itself into an opportunistic pathogen. *Candida*-derived lipase has been identified as potential virulence factor. Even though cellular components of the innate immune response, such as dendritic cells, represent the first line of defense against invading pathogens, little is known about the interaction of these cells with invading *C. parapsilosis*. Thus, the aim of our study was to assess the function of dendritic cells in fighting *C. parapsilosis *and to determine the role that *C. parapsilosis*-derived lipase plays in the interaction with dendritic cells.

**Results:**

Monocyte-derived immature and mature dendritic cells (iDCs and mDCs, respectively) co-cultured with live wild type or lipase deficient *C. parapsilosis *strains were studied to determine the phagocytic capacity and killing efficiency of host cells. We determined that both iDCs and mDCs efficiently phagocytosed and killed *C. parapsilosis*, furthermore our results show that the phagocytic and fungicidal activities of both iDCs and mDCs are more potent for lipase deficient compared to wild type yeast cells. In addition, the lipase deficient *C. parapsilosis *cells induce higher gene expression and protein secretion of proinflammatory cytokines and chemokines in both DC types relative to the effect of co-culture with wild type yeast cells.

**Conclusions:**

Our results show that DCs are activated by exposure to *C. parapsilosis*, as shown by increased phagocytosis, killing and proinflammatory protein secretion. Moreover, these data strongly suggest that *C. parapsilosis *derived lipase has a protective role during yeast:DC interactions, since lipase production in wt yeast cells decreased the phagocytic capacity and killing efficiency of host cells and downregulated the expression of host effector molecules.

## Background

*Candida parapsilosis *is an emerging human pathogen that is currently the second or third most commonly isolated *Candida *species from blood cultures worldwide [1-4]. *C. parapsilosis *typically is a commensal of human skin and is considered to be of low pathogenicity in the setting of intact host barriers. The species is notorious for its capacity to form biofilms on catheters and other implanted devices, for nosocomial spread by hand carriage, and for persistence in the hospital environment [1,3,5]. *C. parapsilosis *is of special concern in critically ill neonates, causing more than one quarter of all invasive fungal infections in low birth weight infants in the UK [6] and North America [7,8], and it is a leading cause of neonatal mortality. In low-birth weight neonates, mortality rates are similar between infants with invasive disease due to *C. parapsilosis *and *C. albicans*, 39 vs. 42%, respectively [6]. Hence, detailed knowledge of *C. parapsilosis *interaction with the host has become urgent. However, host immunity to *C. parapsilosis *infections represents an important, yet understudied area.

Recognition and innate immune response against *Candida *spp. is effected by both professional (eg. macrophages, neutrophils, dendritic cells) [9] as well as semi-professional (eg. epithelial cells) [10] immune cells. The most potent phagocytic cells of the immune system are neutrophils and macrophages, and they are also considered as the prototypical phagocytic cells of pathogenic *Candida *[11]. However, the strategic location of antigen-presenting dendritic cells (DC) at epithelial surfaces and in the skin, the primary sites of *C. parapsilosis *occurrence, places DCs in the first line of defense against invading yeast cells. It has recently been shown that *C. parapsilosis *induces DC fungipod formation [12], which is associated with immune recognition. Importantly the fungipod response is species specific, since the related fungal pathogens *C. tropicalis *and *C. albicans *induce very few and no fungipods, respectively, suggesting significant differences between the response of DCs to different pathogenic *Candida *species. [12]. At present, the role of DCs in *C. parapsilosis *pathogenesis, such as the induction of cytokine gene and protein expression, phagocytosis or fungicidal activity by DCs, is poorly understood.

Although the clinical importance of *C. parapsilosis *is growing, little is known about its virulence factors. Secretion of extracellular hydrolytic enzymes can facilitate disease and lipases have been associated with *C. parapsilosis *virulence [13], however the exact role of this enzyme is still unknown. Putative roles for lipases include the digestion of lipids for nutrient acquisition, adhesion to host cells, synergistic interactions with other enzymes, unspecific hydrolysis, initiation of inflammatory processes by affecting immune cells, and self-defense by lysing the competing microflora. We previously showed that *C. parapsilosis *secreted lipase impacted the capacity of the fungus to grow in lipid rich medium, to produce biofilm, and to survive in macrophages. The production of lipase was essential for *C. parapsilosis *to attach, invade and damage reconstituted oral epithelium, and to invade host tissues in a murine infection model [13]. Concomitantly, we have evaluated the role of Lip8, a key lipase in *C. albicans*, and recapitulated our findings that lipases can be important virulence factors in *Candida *[14].

The aim of our current study is to determine the *in vitro *interaction of human monocyte-derived DCs with wild type and lipase deficient *C. parapsilosis *cells. Because immature and mature DCs (iDCs and mDCs, respectively), show selective responsiveness to different immune and cytokine stimuli we used both cell types in our test system. We have determined that both DC types exert phagocytic and fungicidal activities and produce T-helper (h) 1 type cytokines in response to *C. parapsilosis*. Furthermore we analyzed the role of *C. parapsilosis *lipase by using a lipase deficient mutant and compared the phagocytic capacity and proinflammatory protein production of both DC types.

## Results

### Human monocyte derived dendritic cells internalize lipase deficient mutant yeast cells more efficiently

Although human DCs can phagocytose and eliminate *C. albicans *cells [15], there is little information regarding the outcome of the interactions between DCs and *C. parapsilosis *cells. Therefore, we examined the ability of human monocyte-derived DCs to phagocytose *C. parapsilosis*. For this, iDCs and mDCs were incubated in suspension with unopsonized FITC-labeled live *C. parapsilosis *cells for various periods of time, and phagocytosis was quantified as described in Materials and Methods.

**Figure 1:**
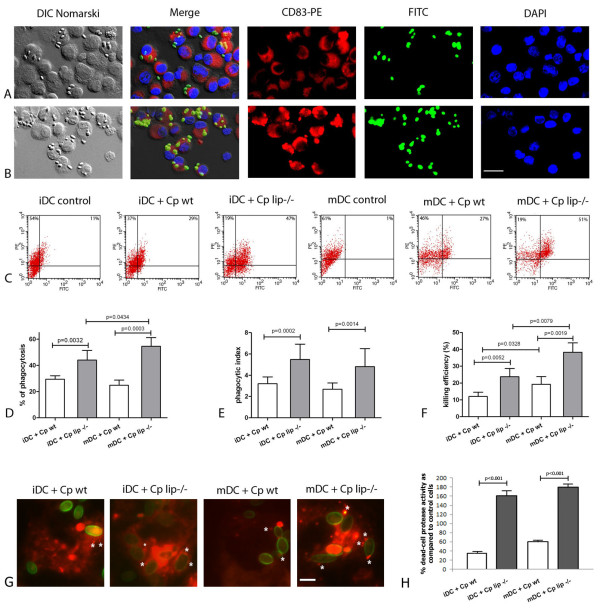
***C. parapsilosis *functionally activates monocyte-derived dendritic cells resulting in increased phagocytosis and killing efficiency**. Panels A and B show representative images of iDCs incubated with unopsonized FITC-labeled wild type (Panel A) and lipase deficient (Panel B) yeast cells at 1 h post-infection. Note that the majority of host cells express CD83, a dendritic cell marker. Panel C shows the FACS plots of DCs infected with wild type (Cp wt) or lipase deficient (Cp lip^-/-^) yeasts at 1 h post-infection. Data on Panels D and E shows the phagocytosis of DCs and are presented as the percent of ingesting cells (percent of DCs containing at least one ingested yeast cell; Panel D) and the phagocytic index (total number of ingested yeast/100 DCs; Panel E). Panel F represents the fungicidal efficiency of DCs, infected with wt or lip^-/- ^*C. parapsilosis*. Panel G shows representative images of DCs incubated with unopsonized FITC-labeled wild type (Cp wt) or lipase deficient (Cp lip^-/-^) yeasts at 1 h post-infection. Lysosomes were visualized by LysoTracker Red. Asterisks show the co-localization of mature lysosomes (red) and phagocytosed yeast cells (green). Data on panel H shows the percentage of the dead-cells as determined by protease activity at 1 h post-infection as compared to the untreated control cells. The data on Panels D-E and H are represented as mean ± SEM of six and two experiments with different donors, respectively. DAPI - 4',6-diamidino-2-phenylindole; wt - wild type; lip^-/- ^- lipase deficient. Scale bars: panels A and B: 20 μm; panel G: 5 μm.

Figure 1A and 1B show that iDCs ingested both wild type and lipase deficient cells after a 1 h co-incubation. Phagocytosis by DCs occurred as early as 30 min (data not shown) after co-culture initiation, and after 1 h 29.4% of iDC and 24.8% of mDC had ingested *C. parapsilosis *wild type cells (Figure 1D). In contrast, more DCs ingested lipase deficient yeast, resulting in phagocytosis rates of 44% (iDC) and 54.6% (mDC) (p value < 0.05) relative to wild type yeast in both DC types (Figure 1D). The phagocytic index data show that phagocytic iDCs internalized an average of 3.2 *C. parapsilosis *wild type yeast cells and mDCs ingested an average of 2.6 yeast cells (Figure 1E). The lack of the lipase production significantly enhanced DC phagocytic index resulting in average indices of 5.7 and 4.6 for iDCs and mDCs, respectively (p value < 0.05) relative to wild type yeast (Figure 1E). To validate and further quantify the phagocytosis percentages of DCs, we also analyzed *C. parapsilosis *phagocytosis by human DCs using FACS. The FACS results correlated to that achieved by microscopy. FACS showed that 29% of iDCs phagocytosed wild type *C. parapsilosis *yeast cells and 47% ingested lipase deficient yeast cells (Figure 1C). Similarly, 27% of mDCs ingested wild type yeast cells and 51% phagocytosed lipase deficient yeast cells (Figure 1C).

### iDCs and mDCs efficiently kill *C. parapsilosis *yeast cells

To assess whether phagocytosis of *C. parapsilosis *cells results in the activation of the antifungal effector machinery in iDCs and mDCs, we performed killing assays using DC co-cultures with *C. parapsilosis *wild type and lipase deficient yeast. The results (Figure. 1F) showed that both iDCs and mDCs were able to efficiently kill *C. parapsilosis *by 3 h post-infection. iDCs and mDCs killed 12% and 13.2% of wild type *C. parapsilosis *yeast cells, respectively. Furthermore, we found that 23% and 38.3% of lipase deficient yeast cells were killed by iDCs and mDCs, respectively, which was significantly higher compared to that of the wild type *C. parapsilosis *(p value < 0.05).

In another series of experiments, we have monitored the viability of DCs after infection with *C. parapsilosis *by measuring the protease activity of the co-cultures. Strikingly, we have found significantly increased number of dead DCs following infection with lipase deficient yeasts compared to uninfected DCs. Increased numbers of dead DCs were present as early as 1 h post-lipase deficient infection (Figure 1H) with only ~10% of DCs remaining viable 24 h post-infection (data not shown). In contrast, DCs infected with wild type yeast cells showed decreased protease activity after 1 h of co-incubation (Figure 1H) with ~50% of DCs still viable at 24 h post-infection. We have obtained similar results when using Trypan blue labeling (data not shown).

Numerous species of the *Candida *genus form pseudohyphae as an effort to avoid killing by phagocytic cells. Our data demonstrate that DCs less efficiently kill lipase deficient compared to wild type *C. parapsilosis *and suggest that wild type yeast cells, at least partially, escape DC immune response. A possible escape mechanism could be pseudohyphae formation. We have monitored the pseudohyphae formation of *C. parapsilosis *in DC-fungi co-culture and determined that *C. parapsilosis *does not form pseudohyphae in our model (Figure 1A, B and data not shown).

Another mechanism by which pathogens modify the immune response of the host is altering lysosome maturation. In order to test if *C. parapsilosis *lipase decreases the phago-lysosome maturation, we have performed labeling with LysoTracker Red, a weakly basic amine that selectively accumulates in acidic compartments such as lysosome. We have observed lysosome maturation in both DC types after infection with wild type and lipase deficient yeast cells (Figure 1G), but there was a decreased number of mature lysosomes in both iDCs and mDCs infected with wild type yeast (Figure 1G).

### Production of IL-1α, IL-6, TNFα, and CXCL8 by iDCs and mDCs exposed to wild type or lipase deficient *C. parapsilosis*

The outcome of encounters between antigen-bearing APCs and naive T cells depends, in part, on the nature of the proinflammatory proteins released locally by the APCs. Proinflammatory cytokines and chemokines, such as IL-1α, IL-6, TNFα, and CXCL8, secreted by various cell types play a fundamental role in attracting neutrophils and T cells to the place of skin infection. Therefore, we determined the pattern of the production of the above mentioned four molecules in DCs exposed to wild type or lipase deficient *C. parapsilosis *by monitoring gene expression and protein secretion using qualitative real-time (QRT)-PCR, cytokine-specific ELISAs, and Luminex Fluorokine Multianalyte Profiling (MAP) assays.

**Figure 2:**
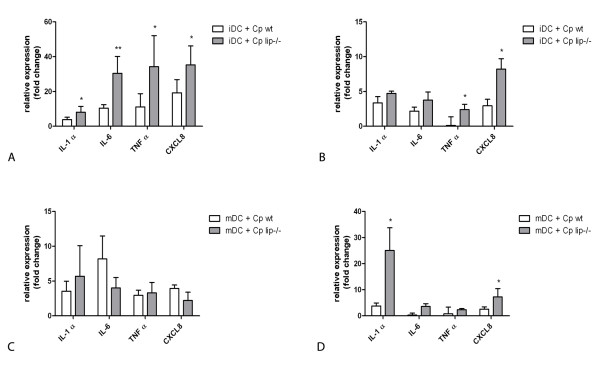
***C. parapsilosis *induces the expression of proinflammatory cytokines and chemokines in DCs**. Quantitative reverse transcriptase polymerase chain reaction (QRT-PCR) analysis of IL-1α, IL-6, TNFα and CXCL8 gene expression in iDCs (Panels A and B) and mDCs (Panels C and D) at 1 h (Panels A and C) and 24 h (Panels B and D) post-infection. DCs were infected with wild type (white columns) or lipase deficient (grey columns) *C. parapsilosis*. Expression levels were normalized and compared to the 18S rRNA and the fold change value was calculated using the ΔΔC_T _method. All measurements were preformed in duplicate for each experiment with at least three biological replicates. * p < 0.05, ** p = 0.002; wt - wild type; lip^-/- ^- lipase deficient

For gene expression studies, cells were harvested at 1 and 24 h post-infection in order to monitor the early and late effects of the infection, respectively. QRT-PCR results revealed that the expression of nearly all of the four proinflammatory genes was significantly higher upon infection with *C. parapsilosis *cells in comparison to the non-stimulated DC populations (p < 0.05), while the expression of TNFα of iDCs infected with wild type yeast cells and IL-6 of mDCs were not increased significantly (Figure 2). Although, IL-1α transcripts were similarly elevated in iDCs at 1 h post-infection with either wild type or lipase deficient *C. parapsilosis*, the increase was significantly greater with the lipase deficient yeast cells (p < 0.05) (Figure 2A). At 24 h, the expression levels with either type of *C. parapsilosis *were similarly increased (Figure 2B). In comparison, mDCs stimulated with lipase deficient cells did not show statistically significant upregulation of IL-1α transcript at 1 h relative to wild type, however the mRNA level increased by almost 35 fold at 24 h (p < 0.05). The IL-6 gene was 30 fold upregulated in iDCs infected with lipase deficient cells compared to wild type yeast at 1 h post-infection (p = 0.002), although there were no differences at 24 h or during infection of mDCs. Interestingly, the TNFα transcript progressively diminished upon exposure to wild type yeast cells, whereas it was upregulated in iDCs infected with lipase deficient yeast cells. Lipase deficient yeast induced significantly higher CXCL8 gene expression at both time points in iDCs (p < 0.05), whereas mDCs increased CXCL8 mRNA levels only at 24 h post-infection (p < 0.05).

For protein measurements, cell culture supernatants were collected at 24 and 48 h post-infection in order to allow protein translation to occur. We detected significantly higher amounts of IL-1α in co-cultures of lipase deficient cells and iDC at 24 h (p value < 0.05), but this difference was not significant at 48 h (Table 1). In contrast, mDCs infected with lipase deficient yeast secreted significantly more IL-1α protein at both time points (p value < 0.05) (Table 2). Consistent with the gene expression, we detected high levels of secreted IL-6 in both iDCs (Table 1) and mDCs (Table 2) at 24 and 48 hours. Similarly, an elevated secretion of TNFα occurred in response to lipase deficient cells at both time points with iDCs (Table 1); however, mDCs produced more TNFα only after 24 h (Table 2). Comparable levels of CXCL8 were measured at 24 h and 48 h after exposure to wild type or lipase deficient cells by both DC populations (Table 1 and 2). These results indicate that, upon exposure to *C. parapsilosis *wild type or lipase deficient yeast, iDCs and mDCs differentially produce IL-1α, IL-6 and TNFα.

**Table 1:** The profile of proinflammatory cytokine and chemokine secretion of iDCs in response to *C. parapsilosis*

	iDC (24 h)		
**(pg/ml)**	**unstimulated**	***Cp *wt**	***Cp *lip-/-**

**IL-1 α**	9.38^† ^(8.20-11.19)	10.01 (8.34-11.17)	23.60^# ^(19.88-26.74)
**IL-6**	175.77 (48.34-252.62)	3059.61 (1689.8-5880.12)	5636.54^# ^(2792.25-7915.07)
**TNF α**	74.36 (55.71-115.78)	624.47 (522.57-736.08)	2836.59^# ^(2822.29-3147.02)
**CXCL8**	794.23 (162.80-1226.77)	3622.8 (2047-5297.31)	3023.9 (1226.41-5297.31)

	**iDC (48 h)**		

**(pg/ml)**	**unstimulated**	***Cp *wt**	***Cp *lip-/-**

**IL-1 α**	7.85 (5.05-12.31)	15.45 (8.34-21.56)	22.14 (19.88-26.74)
**IL-6**	3573.23 (3201.12-4752.01)	5238.9 (3767.13-6082.85)	6968.16^# ^(5398-8938.58)
**TNF α**	154.92 (115.71-194.82)	2342.12 (649.76-4333.62)	3947.27^# ^(2433.01-5393.78)
**CXCL8**	1103.05 (656.02-1473.77)	1615.33 (942.11-1756.85)	1824.31 (1226.41-2491.06)

n = 8 independent blood donors

Immature dendritic cells were stimulated with *C. parapsilosis *wild type (*Cp *wt), lipase deficient (*Cp *lip-/-) cells or left unstimulated. Secretion of IL-1α, IL-6, TNFα or CXCL8 by iDCs was determined by Luminex analyzer or ELISA at 24 h and 48 h post-infection. †: medians (interquartile ranges) # p < 0.05

**Table 2:** The profile of proinflammatory cytokine and chemokine secretion of mDCs in response to *C. parapsilosis*

	mDC (24 h)		
**(pg/ml)**	**unstimulated**	***Cp *wt**	***Cp *lip-/-**

**IL-1 α**	21.90^† ^(6.64- 70.46)	241.71 (19.78- 366.12)	487.97^# ^(110.80- 548.77)
**IL-6**	159.26 (38.75- 226.87)	3934.41 (2481.7-6316.06)	6535.23^# ^(3122.14-9215.14)
**TNF α**	99.51 (58.12-158.89)	1724.67 (736.08-2859.76)	3454.13^# ^(2934.29-4139.50)
**CXCL8**	1632.81 (1358.45-2897.26)	3420.32 (3268-6563.96)	2657.64 (1846.33-3076.52)

	**mDC (48 h)**		

**(pg/ml)**	**unstimulated**	***Cp *wt**	***Cp *lip-/-**

**IL-1 α**	22.97 (11.17-40.30)	35.58 (11.19-68.98)	126.87^# ^(59.90-198.21)
**IL-6**	4364.11 (4025.97-5410.58)	5873.19 (4767.13-7510.32)	7988.22^# ^(6119.10-9893.27)
**TNF α**	124.92 (74.93-163.21)	3456.54 (1628.19-5686.98)	4345.39 (2694.78-5426.10)
**CXCL8**	2223.11 (898.14-4978.58)	2605.43 (1254.21-5297.94)	2392.44 (1226.74-5394.56)

n = 8 independent blood donors

Mature dendritic cells were stimulated with *C. parapsilosis *wild type (*Cp *wt), lipase deficient (*Cp *lip-/-) cells or left unstimulated. Secretion of IL-1α, IL-6, TNF-α or CXCL8 by iDCs was determined by Luminex analyzer or ELISA at 24 h and 48 h post-infection. †: medians (interquartile ranges) # p < 0.05

## Discussion

The phagocytic capacity of macrophages and dendritic cells is an important feature during microbial infection, because the outcome of the interaction of phagocytic cells with fungal pathogens influences the susceptibility of the host to the infection [16,17]. In this work we demonstrate that the emerging fungal pathogen *C. parapsilosis *can be efficiently phagocytosed and killed by human monocyte derived dendritic cells. Our results showed that after 1 h co-incubation 29.4% of iDC and 24.8% of mDC had ingested *C. parapsilosis *wild type cells. Interestingly, in a comparable study, approximately 60% of a given iDC population phagocytose *C. albicans *[9] thus, *C. parapsilosis *cells induce less phagocytosis in comparison to *C. albicans*. In addition, we also observed that lipase deficient *C. parapsilosis *cells were more efficiently ingested by iDCs and mDCs relative to wild type yeast. The microscopy and FACS results demonstrating avid DC phagocytosis of both wild type and lipase deficient yeast is consistent with an activated phenotype of these host effector cells. Moreover, the enhanced phagocytosis of lipase deficient *C. parapsilosis *by DCs relative to wild type yeast cells suggests that lipase interferes with efficient DC activation.

Dendritic cells are able to kill internalized fungal cells. The *in vitro *infections of DCs resulted in a 12% killing of *C. parapsilosis *wild type cells. This result is comparable with that of *C. albicans *(13.6 ± SD 5.4%) [15]. Moreover, DCs did not kill *C. albicans *cells as efficiently as monocytes or macrophages [15], and the *C. albicans *findings and our results are consistent with the concept that the function of DC is to present candidal antigens to T-cells [18] rather than to eliminate the microorganism. Notably, our data showed a significantly elevated killing capacity of human dendritic cells against lipase deficient *C. parapsilosis *strain. In summary, DCs can effectively phagocytose *C. parapsilosis*, but the capacity to kill the yeast cells is less than that of macrophages [19] and according to our recent results, fungal lipase suppresses the fungicidal activity of DCs.

The mechanisms involved in intracellular pathogenesis are diverse. Among fungi, the most studied intracellular pathogen is *Histoplasma capsulatum*, which is able to impair phagosome-lysosome fusion [20,21]. In the case of *C. parapsilosis *wild type strain, we observed that there is a defect in the maturation of the DC phago-lysosome using lysosomal markers of this process. This finding is in agreement with the related species *C. albicans*, where alterations of phagosome maturation and acidification defects have been described [22,23]. The lipase deficient mutants showed higher co-localization with lysotracker stain, suggesting more frequent phago-lysosome fusion and compartment acidification. In addition, our findings highlight that secreted fungal lipases appear to have a role in the protective mechanisms against the host intracellular killing processes.

The immune system may be activated by the recognition of nonself molecules of infectious agents or by recognition of danger signals that include host molecules released by damaged host cells [24]. It is proposed that the two models are compatible, which may also be the case in our model: both *C. parapsilosis *strains induced the expression of chemotactic molecules, in addition, DCs infected with lipase deficient yeast showed increased cell death which is known to be accompanied by the release of danger signals [25]. Consequently, we propose that DCs infected with lipase deficient yeast cells activate more robust immune response.

Although both wild type and lipase deficient *C. parapsilosis *induced strong, time-dependent activation of pro-inflammatory genes such as IL-1α, IL-6, TNF-α, and CXCL-8 in both DC types, lipase deficient yeast induced significantly higher gene expression of effector molecules. Since locally produced chemotactic factors are presumed to mediate the sequence of events leading to the infiltration of immune cells at inflammatory sites, local expression of pro-inflammatory mediators after contact with *C. parapsilosis *could have an initiator role in the attraction of additional immune cells to the sites of infection. This is supported by the fact that CXCL8 is one of the most potent neutrophil chemoattractants [26] that affects not only the recruitment of neutrophils into the tissues but also modulates the ability of these neutrophils to cross epithelial barriers and to kill pathogens. In addition, TNF-α enhances the fungicidal properties of neutrophils, promotes the adhesion of immune to endothelial cells and acts as a danger signal. Corresponding to this finding, we found that DCs infected with lipase deficient yeast cells displayed increased protease activity, which accompanies cell death and the release of danger signals. Finally, TNF-α, IL-1α and IL-6 are also implicated in the induction of antimicrobial peptide expression in epithelial cells [27]. Taken together, the secretion of pro-inflammatory mediators and the release of danger signals by DCs as a response to *C. parapsilosis *may play a crucial role in the recruitment of immune cells into the sites of infection.

## Conclusions

Our work shows that *C. parapsilosis *activates monocyte-derived DCs, as demonstrated by increased phagocytosis and killing of yeast cells and proinflammatory protein secretion. Moreover, we found that DCs infected with lipase deficient *C. parapsilosis *are functionally more potent relative to DCs infected with wild type yeast cells, which suggests that lipase interferes with DC activation. This finding was unexpected because lipases of other pathogenic microorganisms are considered to be inducers of immune response, consequently one would have predicted a decreased activation phenotype in response to lipase deficient *C. parapsilosis*. The fact that this was not the case appears to result, at least in part, the DC activation is suppressed by the *C. parapsilosis *lipase. Further studies will be required to identify the defective anti-*C. parapsilosis *effector mechanisms that increase susceptibility to invasive candidiasis and to determine how *C. parapsilosis *lipase represses immune activation.

## Methods

### Fungal Strains and culture conditions

*Candida parapsilosis *GA1 and lipase deficient (Δ*Cplip1*-Δ*Cplip2*/Δ*Cplip1*-Δ*Cplip2*::*FRT*) strains [13] were maintained at -80°C in 35% glycerol. If not mentioned otherwise, the cells were grown in YPD (1% yeast extract, 2% bactopeptone, 2% glucose).

### Monocyte isolation and dendritic cell differentiation

Human peripheral blood mononuclear cells (PBMCs) were isolated from buffy coat blood samples from healthy donors by Ficoll Paque Plus (GE Healthcare) density gradient centrifugation. Monocytes were isolated by adherence on tissue culture plastic plates. Immature dendritic cells were prepared by culturing monocytes for five days with 1000 U/ml human recombinant granulocyte-macrophage colony stimulating factor (GM-CSF; Sigma) and 1000 U/ml human recombinant interferon-α (IFN-α; Sigma) in RPMI-1640 medium (Gibco) complemented with 10% heat-inactivated FBS (Gibco) and 1% penicillin/streptomycin solution (Gibco) in 6-well tissue culture plate (Sarstedt). Mature dendritic cells were obtained from immature dendritic cells by stimulation with 10 ng/ml recombinant TNFα (R&D Systems) for 24 hours.

### *In vitro *infection

For infections, iDC and mDC cells were co-incubated with *C. parapsilosis *cells at effector-to-target ratios of 1:5 in six-well plates. Samples were incubated for various time at 37°C and 5% (v/v) CO_2_. For gene expression studies DCs were harvested after 1 h and 24 h co-incubations, for cytokine measurement supernatants were collected after 24 h and 48 h.

### Killing assays

Co-cultures of the DCs and *C. parapsilosis *were performed according to our described protocol [13] with some modifications. Briefly, *C. parapsilosis *cells were grown overnight, washed three times in PBS, counted using a hematocytometer, and suspended in RPMI-1640 medium (Gibco). The cells were then co-incubated with DCs as described above. As a control, the same number of *C. parapsilosis *cells were inoculated in the RPMI-1640 medium (Gibco) complemented with 10% heat-inactivated FBS (Gibco) and 1% penicillin/streptomycin solution (Gibco) with no effector cells. The wells were then incubated at 37°C for 3 h, and washed three times with PBS to remove nonadherent *Candida *cells. Yeast cells were liberated from DCs by forcibly disrupting the DCs through pipetting them in distilled water for 2 min. The yeast cells were collected, counted, and serially diluted prior to being plated. Cells were plated in YPD agar and incubated for 3 days at 30°C. The killing efficiency was calculated by normalizing the number of CFU (colony forming unit) counted from the DC infected wells to the total number of CFU of *C. parapsilosis *detected from the control wells, and multiplied by 100 for percentage.

### Phagocytosis assays

Infections were performed as described above and the phagocytosis was monitored by fluorescent microscope after 1 h of co-incubation. Briefly, DCs were treated with FITC-labeled *C. parapsilosis *wild type or homozygous lipase deletion mutant for 1 h. Cells were then trypsinized by using TrypLe Express (Gibco), and washed with PBS. The fluorescence of extracellular yeasts was quenched with 0,4% Trypan blue solution. In some experiments labelling with calcofluor white (0,1 ng/ml (w/v)) was also performed in order to define non-phagocytosed yeast cells (data not shown). After two washes with PBS, cell suspensions were loaded up in each cuvette of a cytospin (Cellspin I, Tharmac). The cells were collected at 600 rpm for 6 minutes and then fixed in PBS with 4% paraformaldehyde for 15 min. The samples were then permeabilized with PBS containing 1% Triton-X (Sigma) for 30 minutes and blocked in PBS containing 1% BSA for 20 minutes. Samples were incubated with 1:10 dilution of phycoeritrin (PE) conjugated anti-CD83 antibody (Life Technologies) in PBS containing 1% BSA and 0.1% Triton-X for 1 h and washed three times with PBS for 5 min each. Negative controls consisted of incubation with isotype matched control (Life Technologies). Finally, samples were washed with PBS containing 4',6-diamidino-2-phenylindole (DAPI) and mounted in Citifluor mounting media (Citifluor Ltd.). Samples were analyzed using epifluorescent illumination of the Axiovision Z1 Fluorescent Microscope (Zeiss) and images recorded by Axiovision software. The percent of phagocytosis was the ratio of the number of DCs that ingested yeast to the total number of DCs multiplied by 100. Phagocytic index was the ratio of the number of intracellular yeast cells to the number of DCs which phagocytozed at least one yeast cell. The number of total DCs, DCs containing yeast cells and ingested *C. parapsilosis *cells were determined from ten individual fields.

### Flow cytometry analysis

Treatment and harvesting of DCs with FITC-labeled *C. parapsilosis *strains was performed as described above. The fluorescence of extracellular yeasts was quenched with 0,4% Trypan blue solution. Cells were washed twice with FACS buffer (2% FBS and 0,5 mM EDTA in PBS). Cells were then incubated with 1:10 dilution of phycoeritrin conjugated anti-CD83 antibody or an isotype matched control (Life Technologies) for 30 minutes at 4°C. Cells were fixed with FACS fix solution (2% FBS, 0,5 mM EDTA and 4% paraformaldehyde in PBS) and analyzed on a FACS Calibur Flow Cytometer (Becton Dickinson) using CellQuest Pro software.

### Lysosome maturation assays

Infections were performed as described above and lysosome maturation was monitored by fluorescent microscopy after 1 h of co-incubation. Briefly, DCs were treated with wild type or a homozygote lipase deletion mutant FITC-labeled *C. parapsilosis*. After 1 h co-incubation the cell culture media was replaced by fresh media supplemented with 50nM LysoTracker Red (Life Technologies) and incubated for additional 45 minute. Cells were then spun and mounted as described in phagocytosis assay section. Samples were analyzed using epifluorescent illumination of the Axiovision Z1 Fluorescent Microscope (Zeiss) and images recorded by Axiovision software.

### Cell viability assays

Treatment and harvesting of DCs with *C. parapsilosis *strains was performed as described above. After 1 and 24 hours co-incubation, cells were transferred into 96-well U-bottom opaque plate (Greiner). Dead-cell protease activity was measured using Cyto Tox-Glo Cytotoxicity Assay (Promega) following the manufacturer's instructions. Luciferase activity was measured by microplate luminometer (LUMIStar Optima, BMG Labtech).

### Quantitative reverse transcriptase polymerase chain reaction (QRT-PCR)

Total RNA was extracted from DCs using RNeasy Plus Mini Kits (Qiagen) according to the manufacturer's instruction. The quality and quantity of the extracted RNA was determined using NanoDrop (Thermo Scientific), Qubit (Life Technologies) and Bioanalyzer (Agilent) measurements. cDNA was synthesized from 150ng of total RNA by using High Capacity RNA to cDNA Kit (Life Technologies) on a Veriti Thermal Cycler (Life Technologies). TaqMan technology based real-time quantitative PCR was used to quantify the relative abundance of each mRNA (StepOne Plus Real-Time PCR System; Life Technologies). For this, specific exon spanning gene expression assays were used for IL-1α (Hs00174092_m1), IL-6 (Hs00174131_m1), TNFα (Hs00174128_m1), CXCL8 (Hs00174103_m1) and 18S rRNA (Hs99999901). As controls, we used the reaction mixtures without the cDNA. All measurements were preformed in duplicate for each experiment with at least three biological replicates. The ratio of each mRNA relative to the 18S rRNA was calculated using the ΔΔC_T _method.

### Measurement for secreted cytokine levels

Harvested cell culture supernatants were centrifuged and the concentrations of secreted IL-1α, IL-6 and TNF-α were measured by Fluorokine Multianalyte Profiling (MAP) Kits (R&D Systems, Inc.) on a Luminex analyzer (Luminex Corp.), according to the manufacturer's instruction. CXCL8, IL-1α, IL-6 and TNFα proteins were also measured using the Quantikine human immunoassay kits (R&D Systems, Inc.) following the manufacturer's instructions. We used serial dilutions of the respective recombinant human proteins for generating standard curves. The optical density of the wells was determined using a microplate reader (FLUOstar Optima, BMG Labtech) set to 450 nm with a wavelength correction set to 540 nm.

### Statistical analysis

The significance of differences between sets of data was determined by Newman-Keuls test or ANOVA according to the data by using GraphPad Prism version 5.02 for Windows (California, USA).

## Competing interests

The authors declare that they have no competing interests.

## Authors' contributions

FK, TN and AG carried out the phagocytosis and QRT-PCR studies, participated in the protein measurement experiments. ZSH, IN and AG participated in the infection studies. IN and AG participated in the design of the study and performed the statistical analysis. IN, CV and AG conceived of the study, and participated in its design and coordination and helped to draft the manuscript. All authors read and approved the final manuscript.
